# Jameson’s Extraordinary Case of Twins

**Published:** 1842-10-29

**Authors:** William Jameson

**Affiliations:** Assistant Surgeon to Mercer’s Hospital, &c.


*Extraordinary Case of Twins.* By William Jameson, M.D., Assistant Surgeon to Mercer's Hospital, &c.—On the evening of the 3d April last, I was called to Fitzwilliam Place, to see Mrs. R., a lady about 30 years of age, the mother of four living children, in consequence of severe pain experienced through the abdomen, which recurred at uncertain intervals, lasting generally about five minutes at a time.

These pains commenced in the morning after breakfast, but in consequence of their becoming more severe, and dreading inflammation, she sent for me.

When I arrived she was in bed, her pulse a little accelerated, tongue clean, no tenderness discoverable on pressure in any part of the abdomen, which felt full, but being seized during the examination with pain, I then distinctly discovered a firm, hard tumor, that reached as high as the umbilicus, but became softer on the subsidence of the pain, and which appeared to me to be a gravid uterus. On applying a stethoscope over the tumor, after some difficulty I thought I heard a placental murmur in the right iliac fossa, but no fœtal heart anywhere, and suggested the possibility of her being with child, and then in labour. This, however, she considered to be impossible, as she had been confined so recently (seven weeks) of a child she was then nursing. A vaginal examination was refused; I found, however, on enquiry, that she had a slight red discharge from the vagina during the latter part of the day, which she considered menstrual.

As I was convinced the tumor was the uterus, and that it was acting to get rid of something or other, I ordered an oil draught, and quitted the apartment in order to explain to the husband my views of the case, viz. that there was some foreign body in the womb, which in all probability would soon be expelled.

While we were thus conversing on the subject, I was hurriedly summoned to my patient, who told me there was something coming from her. On making the examination during the pain I found the head of a small child presenting with the membranes complete, and on the recurrence of another pain, the child, membranes, and placenta, were expelled altogether. I immediately opened the bag containing very little liquor amnii, and found in it a dead male child, at about the sixth month of gestation: shrivelled and dark, but not at all putrid or decomposed, between eight and nine inches long. The cord was small, easily giving way under the fingers: but the placenta appeared to be fully as large as one belonging to a full-grown fœtus, and healthy. Indeed I have often met with a smaller one at the full period. The uterus now contracted well. I placed a binder round the abdomen, administered an opiate, and never saw a more astonished female.

The account I received was as follows. It appeared that she had had four previous confinements without any untoward circumstances, each labour being generally complete in a few hours. That the youngest of them was a boy then only seven weeks old, having been born on the 13th February last, which she was nursing. That she had been attended by a midwife at her residence in the county of Wicklow (her usual medical attendant being out of the way when sent for;) that on that occasion her labour went on much the same as usual, having been completed in four hours; that the after-birth was thrown off about ten minutes after the birth of the child, without any subsequent hæmorrhage; and that she recovered and was able to go about her domestic arrangements as soon as on former occasions; but she remarked that her size had not much decreased, which she imputed to bad swathing of the abdomen. That her general health was good; had as much milk as usual, and the only thing that annoyed her was her size, which she considered a natural consequence of her situation, as she always got fat while nursing.

That the last time she menstruated was in the latter end of the month of April, 1841, and as she had been confined on the 13th of February last, forty-two weeks must consequently have elapsed between the last period of menstruation and the birth of the child.

The above case I consider one of great interest, whether viewed, in the first place, as regards the size of the child, it being as large as a child at the sixth month; the circumstance of its having been carried forty-nine weeks (within three weeks of twelve months) after the last act of menstruation, and remaining for such a length of time after its growth was arrested in utero without becoming decomposed; the labour pains ceasing after the birth of the first, and no inconvenience arising from, or suspicion of anything remaining in the uterus after that accouchement. All these are points, I say, of great interest. Secondly, to view it as a case of superfœtation, or as one of common twins, in which the first was expelled under ordinary circumstances at the full period; then all labour pains have ceased, and an ignorant woman being in attendance, who did not know whether a second child remained behind or not, from the feel of the abdomen, while a second did not only exist, but was carried for seven weeks afterwards without any suspicion of its existence.

I am fully aware that many practitioners have met cases (a similar one of which occurred with myself,) of abortion having taken place in twins, where one ovum was expelled, and that notwithstanding the female has gone on progressively increasing in size, and in a few months, as the case might be, brought forth a living child at the full period. Other instances again, of twin cases, in which one child has gone on to the full period, and been healthy and full grown, while its companion in utero has been blighted at a very early period (a beautiful preparation illustrative of which we have preserved in Coombe Hospital Museum,) and been expelled at the same labour, giving rise to the idea of superfœtation, or of a woman having conceived again while she was pregnant, from the circumstance of the child not being putrid, some erroneously imagining that a dead child cannot be carried for any length of time without being found in that state.

But I am not aware of any instance in which a woman was pregnant of twins (evidently the case here,) where, at the full period, labour set in, and went on healthily, expelling one child alive, while another, dead, was retained and held in the womb for seven weeks afterwards.

The retention of the placenta is also a matter deserving of attention; why was there not hæmorrhage after the birth of the living child, inasmuch as the presence of the unexpelled contents prevented uterine contraction to have gone on sufficiently for that purpose. The only answer I should give to this is, that the ovum remaining in the womb, and of a sufficient size, must have pressed against the surface to which the other placenta was attached, and in that way have saved the patient.—*Dublin Journ. of Med. Science.*

